# Interval Estimation for Age-Adjusted Rate Ratios Using Bayesian Convolution Model

**DOI:** 10.3389/fpubh.2019.00144

**Published:** 2019-06-05

**Authors:** Yunyun Jiang, Andrew B. Lawson, Li Zhu, Eric J. Feuer

**Affiliations:** ^1^Department of Epidemiology and Biostatistics, George Washington University, Washington, DC, United States; ^2^Department of Public Health Sciences, Medical University of South Carolina, Charleston, SC, United States; ^3^Surveillance Research Program, Division of Cancer Control and Population Sciences, Statistical Research and Applications Branch, National Cancer Institute, National Institutes of Health, Bethesda, MD, United States

**Keywords:** spatial correlation, rate ratio, Bayesian statistics, BCM model, CAR prior

## Abstract

Spatial correlation raises challenges in estimating confidence intervals for region specific event rates and rate ratios between geographic units that are nested. Methods have been proposed to incorporate spatial correlation by assuming various distributions for the structure of autocorrelation patterns. However, the derivation of these statistics based on approximation may have to condition on the distributional assumption underlying the data generating process, which may not hold for certain situations. This paper explores the feasibility of utilizing a Bayesian convolution model (BCM), which includes an uncorrelated heterogeneity (UH) and a conditional autoregression (CAR) component to accommodate both uncorrelated and correlated spatial heterogeneity, to estimate the 95% confidence intervals for age-adjusted rate ratios among geographic regions with existing spatial correlations. A simulation study is conducted and a BCM method is applied to two cancer incidence datasets to calculate age-adjusted rate/ratio for the counties in the State of Kentucky relative to the entire state. In comparison to three existing methods, without and with spatial correlation, the Bayesian convolution model-based estimation provides moderate shrinkage effect for the point estimates based on the neighbor structure across regions and produces a wider interval due to the inclusion of uncertainty in the spatial autocorrelation parameters. The overall spatial pattern of region incidence rate from BCM approach appears to be like the direct estimates and other methods for both datasets, even though “smoothing” occurs in some local regions. The Bayesian Convolution Model allows flexibility in the specification of risk components and can improve the accuracy of interval estimates of age-adjusted rate ratios among geographical regions as it considers spatial correlation.

## Introduction

The ratio of age-adjusted rates is a common measure in public health for comparing rates between certain population groups or geographic units. The rate ratio comparing rates in a set of geographic units with an area considered to be “standard” is especially of interest to public health policy stake-holders on program planning and resource allocation. The key aspect of RR estimation between geographic units is the accommodation of spatial correlation, and the overlap of each region with the overall study region. There is a necessity to consider both sources of correlation into the interval estimation for rates. Failing to account for spatial correlation leads to an underestimated variability in the point estimate with lower statistical power. Thus far, the approximation method based on well-known statistical distributions (Gamma and F interval) (1, 2) has been proposed to address the correlation between sub-region and overall regions. Further, Zhu et al. (3) developed a method to incorporate the spatial autocorrelation across regions into the confidence interval (CI) by assuming the structure of specific autocorrelation patterns which follows an exponential distribution.

The Bayesian convolution model (BCM), which includes a linear combination of an uncorrelated random effect and a random effect correlated spatial heterogeneity, was first proposed by Besag et al. (4) as a disease mapping technique to model the within/between region variability of the event rate. The variability can be decomposed into both correlated and uncorrelated random effects for spatial heterogeneity. As compared to other methods, the advantage of BCM is that inferences are made only through observed data without over-specifying the asymptotic distributions. In addition, for BCM model, the spatial correlation structure is determined by the first order spatial effect rather than the actual distance between regions, and that provides a smoothing effect for the relative risk estimates in individual areas toward the local average. (5) BCM has been found to yield the best recovery of true risk under a variety of true risk scenarios (6).

The first papers that tackled CI for age-adjusted rates (AAR) and rate ratios (RR) were published by Fay and colleagues (7, 8) based on the assumption that AARs follow a gamma distribution. Tiwari et al. (1) expanded the algorithm to calculate the RR of a sub-region and its parent region excluding the sub-region itself. This algorithm does not consider overlap between the two regions that the rate ratio is calculated for (i.e., a sub-region and a parent region including the sub-region). It is labeled the “Direct” method in the rest of this paper.

To take into account the overlap between a sub-region and its parent region, Tiwari and colleagues (2) developed an extension to the earlier method that performed well in intensive simulation studies. The approach derived 95% confidence intervals for RR of *R*_*i*_ and *R*_Ω_ based on F-approximations as well as normal approximations. Through intensive simulations, they found that F-intervals are often more conservative for rare cancer sites; for moderate and common cancers, both intervals perform similarly. This approach is labeled “Overlap” in the rest of this paper to indicate that overlap between a sub-region (e.g., county in a state) and its parent region (state) is considered.

More recently, Zhu et al. (3) developed an algorithm that considered both overlap between a sub-region and its parent region, as well as the spatial autocorrelation between all regions in the study area. The approach considered a variance-covariance structure of RR that include three parts—variance of the sub-region rates, variance of the parent region rate, and the covariance which contains both overlap of the sub-region and its parent, and spatial autocorrelation between the sub-regions. A parametric form of spatial semivariogram was assumed and estimated, and then the variance-covariance matrix of RR was calculated. The delta method was applied to transform the variance-covariance matrix back to the original scale of RR. This method is labeled “Spatial” in the rest of paper due to the inclusion of spatial autocorrelation between regions. It was pointed out that the “Spatial” method provided substantial improvements over the Direct and Overlap methods by allowing for spatial autocorrelation. When spatial autocorrelation is not strong, the “Spatial” method performs equally as well as the Overlap method.

In this paper we propose a BCM approach and make comparison among the four existing methods. The objective of the study is to 1) explore the usefulness of Bayesian hierarchical convolution model in the cancer registry data with spatial correlations; and 2) compare the interval estimation based on BCM with other approximation methods for the age adjusted rate or RR. The proposed method is labeled as BCM method in the figures and tables presented in this paper.

## Methods

For *I* geographic units and *J* age groups in the study area, let's assume that the data available are *D*_*ij*_, the number of deaths (or new cases), and *n*_*ij*_, the count of the population size from region *i* and age group *j*, then the age-specific rate, *R*_*ij*_, often expressed as number of cases per 100,000 people at risk, is calculated as $R_{ij}=\frac{D_{ij}}{n_{ij}}\times 100,000$. A direct age-adjustment is

(1) $$\begin{array}{l} \begin{array}{l} R_{i}=\overset{J}{\underset{j=1}{\sum }}w_{j}\frac{D_{ij}}{n_{ij}}\times 100,000\\ \;=\overset{J}{\underset{j=1}{\sum }}w_{j}R_{ij} \end{array} \end{array}$$

where *w*_*j*_ is the proportion of population size for age group *j* in the standard population and $\sum_{j=1}^{J}w_{j}=1$. Hence, the AAR is the weighted average of age-specific rates, weighted by the standard population. Let Ω denote the total region of interest, e.g., a whole state where data come from. Then the overall rate for Ω is computed by age adjustment after summing the number of deaths (numerator) and population (denominator) over all the geographic regions, i.e.,

(2) $$\begin{array}{r} \begin{array}{c} R_{\Omega }=\overset{J}{\underset{j=1}{\sum }}w_{j}\frac{\overset{I}{\underset{i=1}{\sum }}D_{ij}}{\overset{I}{\underset{i=1}{\sum }}n_{ij}}\times 100,000\\ \;=\overset{J}{\underset{j=1}{\sum }}w_{j}R_{j} \end{array} \end{array}$$

For the rest of this paper, we use *R*_*i*_, *R*_Ω_, and *D*_*i*_, *D*_Ω_ to denote the random variables for the sub-regional and overall area AAR and count, respectively. The corresponding lower-case letters denote the observed rates or counts, or realizations of the random variables, respectively. It is assumed that the age-specific counts *D*_*ij*_ are independent Poisson random variables with parameters λ_*ij*_, the relative risk of events in area *i* and age stratum *j* as compared to the expected reference rate, and, *D*_*ij*_ ~ *Poisson*(*n*_*ij*_[λ_*ij*_]).

### Direct Method

The “Direct” method refers to the algorithm developed by Tiwari et al. (1). The CIs of RR between *R*_*i*_ and *R*_(−*i*)_ were developed to approximate the rate ratio of *R*_*i*_ to *R*_Ω_, where *R*_(−*i*)_ refers to the AAR of the whole area after deleting region *i*. Since *R*_*i*_ and *R*_(−*i*)_ are linear combinations of independent Poisson random variables *D*_*ij*_, the mean *u*_*i*_, *u*_(−*i*)_, and the variance ${\sigma^{2}}_{i}$, ${\sigma^{2}}_{\left(-i\right)}$ of *R*_*i*_ and *R*_(−*i*)_ are also linear combinations of λ_*ij*_. Applying the delta method, both the mean and the variance of the rate ratio are estimable as the linear combinations of the observed age-specific rates *r*_*ij*_:

(3) $$\begin{array}{r} E\left(\frac{R_{i}}{R_{\left(-i\right)}}\right)\approx \frac{\mu_{i}}{\mu_{\left(-i\right)}} \end{array}$$

(4) $$\begin{array}{r} Var\left(\frac{R_{i}}{R_{\left(-i\right)}}\right)\approx \frac{\sigma_{i}^{2}\mu_{\left(-i\right)}^{2}+\sigma^{2}\mu_{i}^{2}}{n\mu_{\left(-i\right)}^{4}} \end{array}$$

The details of this method can be found at Appendix A of Tiwari et al. (1).

### Overlap Method

The Overlap method is based on the proportional age-distribution assumption (2), i.e., the ratio of the population in a sub-region to that of the parent region is approximately the same across all age-groups. This proportion, denoted as *p*_*i*_, accounts for the overlap in the sub-region's population and that of the parent region. The parent region AAR *R*_Ω_ is approximately a linear combination of the AAR of *R*_*i*_ and *R*_(−*i*)_, i.e., *R*_Ω_ ≈ *p*_*i*_*R*_*i*_+*p*_(−*i*)_*R*_(−*i*)_. Hence

(5) $$\begin{array}{r} \frac{R_{i}}{R_{\Omega }}=\frac{R_{i}/R_{\left(-i\right)}}{p_{i}R_{i}/R_{\left(-i\right)}+p_{\left(-i\right)}} \end{array}$$

where *p*_(−*i*)_ = 1 − *p*_*i*_. Hence, CIs of $\frac{R_{i}}{R_{\left(-i\right)}}$ (derived in the “Direct” method) will lead to those for the rate ratio $\frac{R_{i}}{R_{\Omega }}$. The details of this method can be found in Tiwari et al. (2).

### Spatial Method

The “Spatial” method expands the “Overlap” method to include not only the overlap *p*_*i*_, but also the spatial autocorrelation between regions *i* and *i*′, which is estimated with an exponential semivariogram function. According to the First Law of Geography by Tobler (9), that “everything is related to everything else, but near things are more related than distant things,” it is assumed that the correlation between values measured in two locations decreases with distance. A Kriging technique (10) is applied to the generalized linear models (11, 12) to estimate the covariance between the rates in two locations. To find the variance of rate ratio R_i_/R_Ω_. the logarithm of the rate ratio is considered and

(6) $$\begin{array}{c} Var(\ln(R_{i}/R_{\Omega })=Var(\ln\;R_{i})+Var(\ln\;R_{\Omega })\\ -2Cov(\ln\;R_{i},\ln\;R_{\Omega }) \end{array}$$

In the equation above, the variance of the logarithm of the AARs can be estimated using the delta method and the variance of the AARs, and the covariance term is estimated using the assumed exponential semivariogram function. The resulting variance of rate ratio R_i_/R_Ω_ can be decomposed into four components, which represent the variance of the sub-region AAR *R*_*i*_, the variance of the parent region AAR *R*_Ω_, the correlation of *R*_*i*_ and *R*_Ω_ due to population overlap, and the autocorrelation between *R*_*i*_and $R_{i^{\prime }}$ (AAR in another region *i*′).

### Bayesian Convolution Model (BCM)

Under the same assumption that *D*_*ij*_ ~ *Poisson*(*n*_*ij*_λ_*ij*_) and that the counts are conditionally independent given λ_*ij*_, where λ_*ij*_ represents the relative risk of events in area *i* and age stratum *j* as compared to the expected reference rates. We can estimate λ_*ij*_ as:

(7) $$\begin{array}{r} \log\left(\lambda_{ij}\right)=\alpha_{0}+\upsilon_{i}+u_{i} \end{array}$$

where the terms consist of an intercept α_0_, uncorrelated (*v*_*i*_) and correlated (*u*_*i*_) heterogeneity, respectively. The convolution is the spatial random effect defined as ξ_*i*_ = υ_*i*_ + *u*_*i*_, where υ_*i*_ and *u*_*i*_ are used to capture spatially correlated and unstructured extra variation in the model (13). α_0_ represents the baseline log relative risk of disease across study region and age strata. Generally, the uncorrelated and correlated heterogeneity effects are specific for area *i* and age *j*, but in this study, we assume that the heterogeneity effects do not change among age groups and only vary with geographic areas. Additional terms can be included in the definition of the model for the risks as needed. We denote this model as BCM (14), chapter 7. If the model only contains the uncorrelated heterogeneity effect, it is referred to be UH model. Details of the prior specification and posterior inference for BCM model are shown below.

#### Prior Specification

We need to specify prior and hyper-prior for the model parameters. The prior distribution of intercept α_0_ has a normal distribution with mean of 0 and variance of $\sigma_{\alpha }^{2}$ (or precision $\tau_{\alpha }^{}$), the standard deviation follows a uniform distribution:

(8) $$\begin{array}{r} \alpha_{0}\sim N\left(0,\tau_{\alpha }^{-1}\right),\tau_{\alpha }=\frac{1}{\sigma_{\alpha }^{2}},\sigma_{\alpha_{0}}\sim uniform\left(0,100\right) \end{array}$$

The uncorrelated heterogeneity (**v**) has a zero-mean Gaussian prior distribution.

(9) $$\begin{array}{r} \left[v_{i}|\ldots \right]\sim N\left(0,\tau_{v}^{-1}\right),p\left(\text{v}\right)\propto \tau_{v}^{m}\exp\left\{-\frac{1}{2}\tau_{v}\overset{m}{\underset{i=1}{\sum }}v_{i}^{2}\right\} \end{array}$$

The intrinsic conditional autoregression's (autoregressive) improper difference prior distribution (CAR) is assumed for the structured correlated effect **u** (8), and it also follows a singular normal distribution:

(10) $$\begin{array}{r} u_{i}\sim CAR\left(\tau_{u}^{-1},n_{\delta_{i}}\right)or\left[u_{i}|\ldots \right]\sim N\left({\overset{¯}{u}}_{\delta_{i}},\frac{\sigma_{u}^{2}}{n_{\delta_{i}}}\right){ū}_{\delta_{i}} \end{array}$$

ū_δ_*i*__ and $\sigma_{u}^{2}/n_{\delta_{i}}$ are the conditional moments of the intrinsic Gaussian formulation mean and variance, respectively. ${ū}_{\delta_{i}}=\underset{i^{\prime }\in \;\delta_{i}}{\sum }u_{i^{\prime }}/n_{\delta_{i}}$ is the average over the neighborhood of the ith region, $\tau_{u}^{}$ is the precision of the estimation for correlated heterogeneity effect, which is simply the inverse of the already defined variance hyper-parameter. *n*_δ_*i*__ is the number of regions in the neighborhood of i th region.

The hyper-priors for the standard deviation of the intercept is assumed to be uniformly distributed σ_α_0__ ~ *uniform*(0, 100). The hyper-priors for variance parameters for uncorrelated ($\sigma_{\nu }^{2}$) and correlated spatial random effects ($\sigma_{u}^{2}$) are assumed to be inverse gamma distribution IG(k, θ), which has the similar idea to assume a gamma hyper prior for the precision parameters τ_*u*_ and τ_ν_, k and θ are the shape and scale parameters. In our program we assume *k* = 2 and θ = 0.5.

(11) $$\begin{array}{r} \;Prior\;\left(\tau_{u},\tau_{\nu }\right)\;\propto \frac{1}{\Gamma \left(k\right)\theta^{k}}x^{k-1}e^{-\frac{\tau_{u}}{\theta }}\frac{1}{\Gamma \left(k\right)\theta^{k}}x^{k-1}e^{-\frac{\tau_{v}}{\theta }},\\ \text{k}=2,\theta =0.5. \end{array}$$

The posterior distribution based on Poisson likelihood is formulated as

(12) $$\begin{array}{r} P\left(\alpha_{0},u,\nu ,\tau_{u},\tau_{\nu }|D_{ij}\right)=L\left(\text{D}|\text{e},\theta ,\delta \right)p\left(\alpha_{0}|\sigma_{\alpha_{0}}\right)p\left(\nu |\tau_{v}\right)\\ p\left(u|\tau_{u}\right)p\left(\sigma_{\alpha_{0}}\right)p\left(\tau_{v}\right)p\left(\tau_{u}\right) \end{array}$$

$$\begin{array}{r} \;=\overset{I}{\underset{i=1}{\prod }}\overset{J}{\underset{j=1}{\prod }}\left\{\exp\left(e_{ij}\theta_{ij}\right){\left(e_{ij}\theta_{ij}\right)}^{D_{ij}}/D_{ij}!\right\}\\ \;\times \tau_{\alpha_{0}}^{}\exp\left\{-\frac{1}{2}\tau_{\alpha_{0}}\alpha_{0}^{2}\right\}\\ \;\times \tau_{u}^{I/2}\exp\left\{-\frac{1}{2}\tau_{u}\underset{i}{\sum }\underset{i^{\prime }\in \delta_{i}}{\sum }{\left(u_{i}-u_{i^{\prime }}\right)}^{2}\right\}\\ \;\times \tau_{v}^{I/2}\exp\left\{-\frac{1}{2}\tau_{v}\overset{I}{\underset{i=1}{\sum }}v_{i}^{2}\right\}\times \text{prior(}\sigma_{\alpha_{0}}\text{,}\tau_{u},\tau_{v}\text{)} \end{array}$$

#### Posterior Sampling

The posterior distribution is sampled using Markov Chain Monte Carlo (MCMC) algorithm-Gibbs sampler for *u* and Metropolis-Hastings sampler for the other parameters α_0_ and *v*.

#### Prediction of Age Adjusted Rate (Ratios) and Associated 95% Confidence Interval

To obtain the age adjusted measures $AAR_{i}=\overset{J}{\underset{j=1}{\sum }}w_{j}\frac{D_{ij}}{n_{ij}}$ and $AAR=\overset{J}{\underset{j=1}{\sum }}w_{j}\frac{D_{j}}{n_{j}}$, these measures can be calculated from the raw data, based on the methods described by Zhu et al. (3) and Tiwari et al (2). These statistics are referred as raw statistics. The age-adjusted rate for the overall study area is $AAR=\overset{J}{\underset{j=1}{\sum }}w_{j}\frac{D_{j}}{n_{j}}$, where $D_{j}=\underset{i}{\sum }D_{ij}$ and $n_{j}=\underset{i}{\sum }n_{ij}$. The weights are usually pre-defined (or can be calculated separately). The age-adjusted rate in the ith region is $AAR_{i}=\overset{J}{\underset{j=1}{\sum }}w_{j}\frac{D_{ij}}{n_{ij}}$. The age specific expected rate is $e_{ij}=n_{ij}\frac{\overset{I}{\underset{i=1}{\sum }}D_{ij}}{\overset{I}{\underset{i=1}{\sum }}n_{ij}}$. The age adjusted expected rate is $e_{i}=\overset{J}{\underset{j=1}{\sum }}n_{ij}\frac{\overset{I}{\underset{i=1}{\sum }}D_{ij}}{\overset{I}{\underset{i=1}{\sum }}n_{ij}}$.

#### Prediction of Age Adjusted Rate (Ratios) and Associated 95% Confidence Interval

The AARs *R*_*i*_ and *R*_Ω_ can be calculated from the raw data, using formulas (1) and (2). These statistics are referred as raw statistics.

BCM is fitted using MCMC and yields posterior sampled values for the parameters including intercept (α_0_), uncorrelated (*v*_*i*_) and correlated (*u*_*i*_) spatial heterogeneity effect. From that sample we can obtain both posterior mean estimates of λ_*ij*_ and the credible intervals for λ_*ij*_. Once we fit a model to the original data, the predicted counts $D_{ij}^{P}$ are generated under the fitted model from a predictive distribution $D_{ij}^{P}\sim Poisson\left(n_{ij}\lambda_{ij}^{{}^{*}}\right)$ where $\lambda_{ij}^{{}^{*}}$ is a value from the sampler, then there would be a set of $D_{ij}^{P}$s generated for each of the *g* sampler values. The AARs are computed based on these $D_{ij}^{P}$s. For each $D_{ij}^{Pg}$, the predicted count for the *g-*th value of the sampler, the AARs for specific area and the overall area are computed the same way as in formulas (1) and (2). This provides a sample of predicted AARs and RR, from which it is possible to estimate credible intervals.

## Simulations

Simulation studies are conducted to compare the performance across the four methods—“Direct,” “Overlap,” “Spatial,” and the BCM methods. The simulated datasets are generated based on three simulation scenarios presented in Table 1: (1) Convolution model which includes an intercept term, uncorrelated random noise, and correlated spatial heterogeneity. Intercept and the precision parameter for the random noise are fixed. Precision for the spatial heterogeneity random effect varies between small, medium, and large values. This model is referred as T1. (2) Spatial trend model which includes an intercept and linear trend on both latitude and longitude, as well as a random noise effect with a fixed precision parameter (referred as T2). (3) Uncorrelated random effects on county and age in which the precision on age effect is fixed and the precision on the county random effect ranges between large and small values (T3). Values of the parameters are specified so that the relative risk θ_*ij*_ approximately falls in range (0.3, 3.0). For each of the 6 scenarios, 1,000 datasets are simulated with the total count *D* assumed to be 3,000 and 13,000 to mimic rare and common diseases, respectively. Population is set to be the population of the State of Kentucky, the true data example to be introduced in the next section. The “true” rate ratio is defined as the average of the 1,000 simulated datasets. Simulation scenarios for all model settings are assembled based on parameters listed in Table 1.

**Table 1:** Simulation scenarios and parameters.

**Models**	**T1: BCM model**	**T2: common spatial trend**	**T3: random effect on county and age**
Fixed parameters	α_0_ = 0.1, τ_v_ = 100 (precision for noise)	τ_γ_ = 100 (precision for age random effect)	τ_γ_ = 100 (precision for age random effect)
Varying parameters	τ_u_ = 50 (small), 100 (medium), 200 (large)	α_0_ = −3.2, α_1_ = 0.1, α_2_ = 0.32	α_0_ = −0.1, τ_v_ = 10 (small) α_0_ = 0.05, τ_v_ = 200 (large)

The first 100 of the 1,000 simulated datasets are used to evaluate the performance of the four methods. The evaluation criteria include: (1) mean length of 95% interval estimate (CI); (2) coverage probability, measured by the proportion of overlap between 95% intervals from the simulated datasets and the model fitted 95% intervals; and (3) measure of variability, which is the standard deviation of CI length. Only the first 100 simulated datasets are used due to the lengthy computation time in BCM model. It took 4 weeks to run BCM using the 100 datasets for each of the six simulation scenarios and common (Count = 13,000) or rare (Count = 3,000) diseases.

The results of the simulation study are presented in Table 2 (Count = 13,000) and 3 (Count = 3,000). The four methods are compared based on the length of the interval estimates, the coverage probability, and the standard deviation of the intervals. A narrower interval indicates more liberal estimation. Coverage probability is supposed to be close to 95%. Standard deviation of the intervals indicates variation of the interval estimates. Since the data are simulated according to the Kentucky population, the 120 counties in Kentucky are categorized according to the size of the county population. The 40 large counties have an average population of 191 k, the 40 medium counties have an average population of 48 k, and the 40 small counties have an average population of 24 k. Within each simulation scenario, large counties tend to have more liberal interval estimates, with coverage probability closer to 95%, and variation of interval length tend to be small. There is an obvious improvement in the interval estimates from the Direct method to the Overlap method to the Spatial method, especially when the precision of spatial random effect is large compared to medium or small values. Compared to other methods, BCM model has the largest average CI length and variability of the CI length across all simulation scenarios (Tables 2, 3). When the data were simulated under the spatial convolution model with a very precise correlated spatial effect (i.e., large tau), the BCM approach yields the largest coverage probability among all methods. For all simulation scenarios, the length of CI decreases as population size increases. The performance of each method becomes worse as the precision of the parameters decreases.

**Table 2:** Comparison of four methods under 6 data simulation scenarios for total count at 13,000.

**Simulation scenario**			**County pop**	**Direct**			**Overlap**			**Spatial**			**BCM**		
				**CI length**	**Coverage**	**S.D. of CI**	**CI length**	**Coverage**	**S.D. of CI**	**CI length**	**Coverage**	**S.D. of CI**	**CI length**	**Coverage**	**S.D. of CI**
Total Count = 13,000	T1 Spatial convolu-tion	Largeτ*_*u*_*	Large	0.335	0.910	0.035	0.331	0.910	0.035	0.318	0.912	0.037	0.453	0.923	0.048
			Medium	0.538	0.882	0.066	0.536	0.882	0.067	0.513	0.886	0.071	0.698	0.908	0.088
			Small	0.737	0.860	0.102	0.735	0.860	0.102	0.716	0.864	0.104	0.983	0.881	0.132
		Med τ*_*u*_*	Large	0.338	0.912	0.045	0.334	0.911	0.046	0.334	0.911	0.046	0.453	0.915	0.061
			Medium	0.543	0.896	0.082	0.540	0.897	0.082	0.540	0.897	0.082	0.698	0.891	0.107
			Small	0.748	0.872	0.124	0.746	0.872	0.125	0.745	0.872	0.125	0.983	0.850	0.155
		Small τ*_*u*_*	Large	0.345	0.904	0.061	0.341	0.902	0.061	0.341	0.902	0.061	0.453	0.898	0.079
			Medium	0.556	0.888	0.110	0.553	0.888	0.111	0.553	0.888	0.111	0.698	0.858	0.139
			Small	0.761	0.871	0.158	0.760	0.871	0.158	0.760	0.871	0.158	0.983	0.813	0.191
	T2 Spatial trend		Large	0.349	0.739	0.016	0.345	0.744	0.016	0.293	0.782	0.020	0.453	0.664	0.021
			Medium	0.537	0.765	0.037	0.534	0.767	0.037	0.445	0.815	0.040	0.698	0.675	0.049
			Small	0.787	0.717	0.085	0.785	0.718	0.085	0.708	0.749	0.088	0.983	0.682	0.093
	T3 Random effect on county and age	Largeτ*_*v*_*	Large	0.331	0.853	0.019	0.327	0.855	0.019	0.169	0.914	0.046	0.453	0.927	0.028
			Medium	0.530	0.825	0.040	0.528	0.826	0.040	0.303	0.905	0.063	0.698	0.905	0.056
			Small	0.726	0.812	0.075	0.724	0.812	0.075	0.511	0.878	0.095	0.983	0.887	0.100
		Small τ*_*v*_*	Large	0.343	0.911	0.058	0.339	0.911	0.058	0.339	0.911	0.058	0.453	0.782	0.077
			Medium	0.552	0.893	0.098	0.550	0.893	0.098	0.550	0.893	0.098	0.698	0.732	0.125
			Small	0.759	0.886	0.151	0.757	0.886	0.151	0.757	0.886	0.151	0.983	0.711	0.182

**Table 3:** Comparison of four methods under 6 data simulation scenarios for total count at 3,000.

**Simulation** **scenario**			**County pop**	**Direct**			**Tiwari**			**Spatial**			**BCM**		
				**CI length**	**Coverage**	**S.D. of CI**	**CI length**	**Coverage**	**S.D. of CI**	**CI length**	**Coverage**	**S.D. of CI**	**CI length**	**Coverage**	**S.D. of CI**
Total Count = 3,000	T1 Spatial convolu-tion	Large τ*_*u*_*	Large	0.703	0.865	0.097	0.694	0.866	0.098	0.693	0.866	0.098	0.930	0.888	0.126
			Medium	1.142	0.825	0.226	1.137	0.826	0.227	1.136	0.826	0.227	1.458	0.853	0.265
			Small	1.520	0.812	0.374	1.516	0.813	0.375	1.516	0.813	0.375	1.949	0.822	0.467
		Med τ*_*u*_*	Large	0.710	0.875	0.116	0.701	0.876	0.118	0.701	0.876	0.118	0.936	0.856	0.150
			Medium	1.153	0.839	0.249	1.148	0.840	0.250	1.147	0.840	0.250	1.470	0.807	0.292
			Small	1.504	0.819	0.390	1.500	0.820	0.391	1.499	0.820	0.391	1.963	0.784	0.500
		Small τ*_*u*_*	Large	0.724	0.883	0.148	0.715	0.882	0.149	0.713	0.883	0.061	0.939	0.821	0.183
			Medium	1.176	0.850	0.297	1.171	0.850	0.298	1.167	0.851	0.111	1.473	0.767	0.344
			Small	1.542	0.824	0.446	1.539	0.825	0.447	1.536	0.825	0.158	1.975	0.758	0.557
	T2 Spatial trend		Large	0.734	0.742	0.075	0.725	0.747	0.076	0.724	0.747	0.076	0.914	0.671	0.087
			Medium	1.145	0.758	0.178	1.140	0.760	0.178	1.139	0.760	0.178	1.482	0.684	0.212
			Small	1.567	0.729	0.362	1.563	0.730	0.362	1.563	0.730	0.363	1.881	0.703	0.436
	T3 Random effect on county and age	Large τ*_*v*_*	Large	0.722	0.885	0.142	0.713	0.886	0.143	0.711	0.886	0.143	0.930	0.886	0.093
			Medium	1.180	0.845	0.293	1.175	0.845	0.294	1.172	0.846	0.295	1.456	0.838	0.216
			Small	1.499	0.830	0.437	1.495	0.830	0.438	1.493	0.830	0.438	1.952	0.827	0.435
		Small τ*_*v*_*	Large	0.694	0.809	0.068	0.685	0.813	0.069	0.680	0.815	0.074	0.942	0.712	0.176
			Medium	1.125	0.796	0.175	1.120	0.797	0.175	1.112	0.799	0.179	1.483	0.699	0.326
			Small	1.486	0.787	0.331	1.483	0.788	0.332	1.476	0.789	0.334	1.984	0.704	0.546

## Application Examples

Comparison of the four methods was made using cancer incidence data from the National Cancer Institute's Surveillance, Epidemiology and End Results (SEER) Program. SEER collects data on cancer cases from various locations and sources throughout the United States. This program currently collects and publishes cancer incidence and survival data from state and metropolitan level population-based cancer registries covering ~30% of the US population. The data is released through statistical software SEER^*^Stat (15) for the analysis of SEER and other cancer-related databases, such as mortality and county attributes. SEER^*^Stat calculates the non-adjusted and age-adjusted rates (AARs) of cancer incidence or mortality, as well as the rate ratio and the 95% CIs of the RR between selected geographic areas using the Direct method.

**Figure 1:**
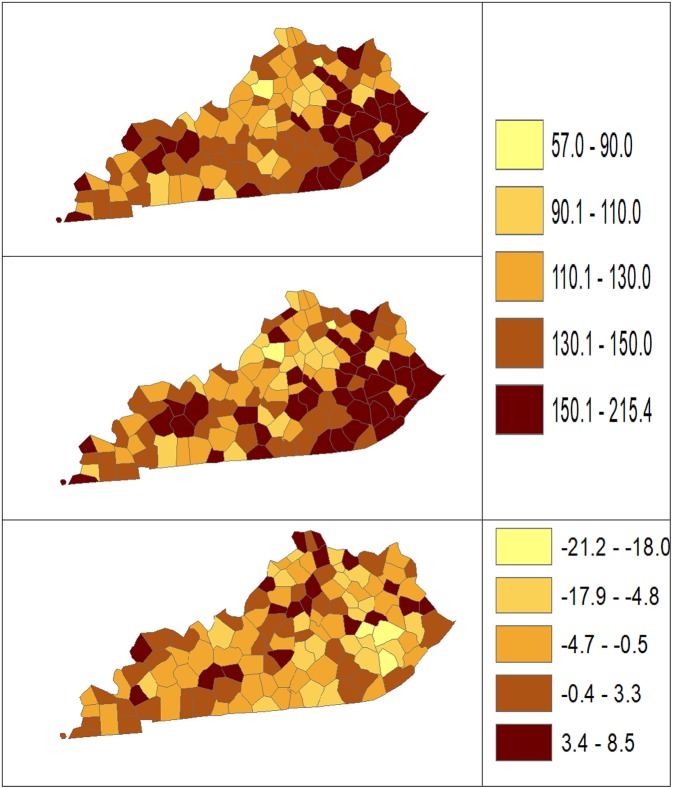
Age-adjusted rate estimated from statistical methods (Direct top vs. BCM middle) and their difference (bottom) using 2006–2010 lung cancer incidence data in Kentucky counties.

**Figure 2:**
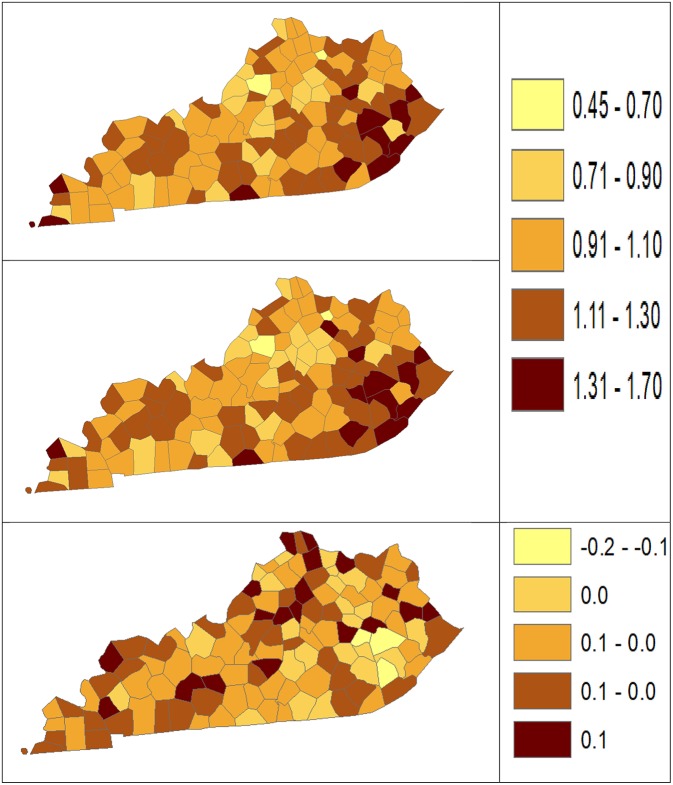
Rate ratio estimated from statistical methods (Direct top vs. BCM middle) and their difference (bottom) using 2006–2010 lung cancer incidence dataset.

**Figure 3:**
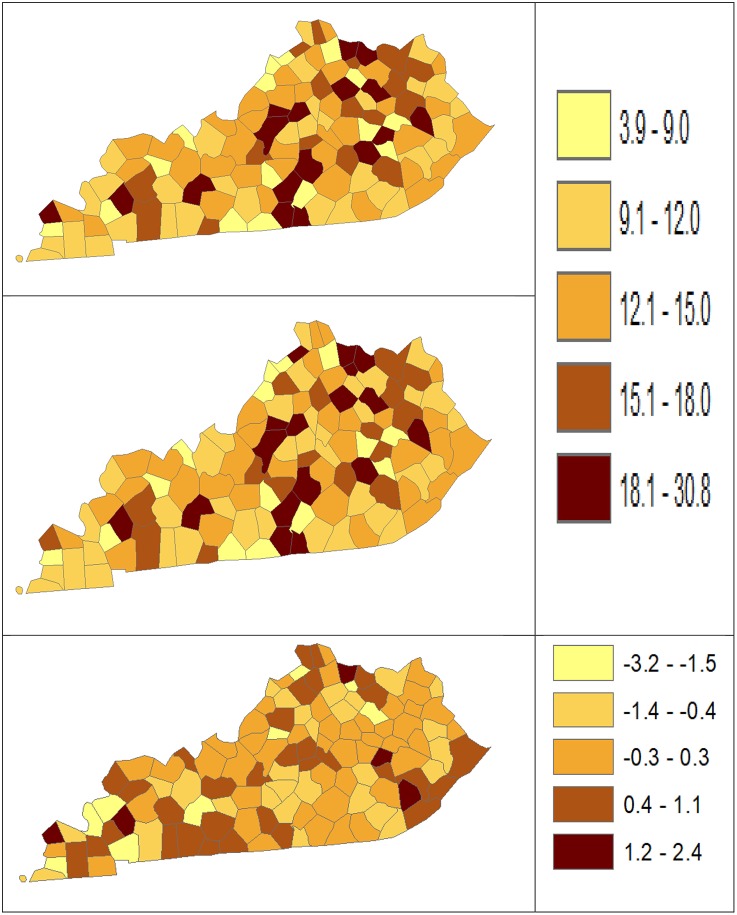
Age-Adjusted Rate estimated from statistical methods (Direct top vs. BCM middle) and their difference (bottom) using 2006–2010 oral cancer incidence dataset.

**Figure 4:**
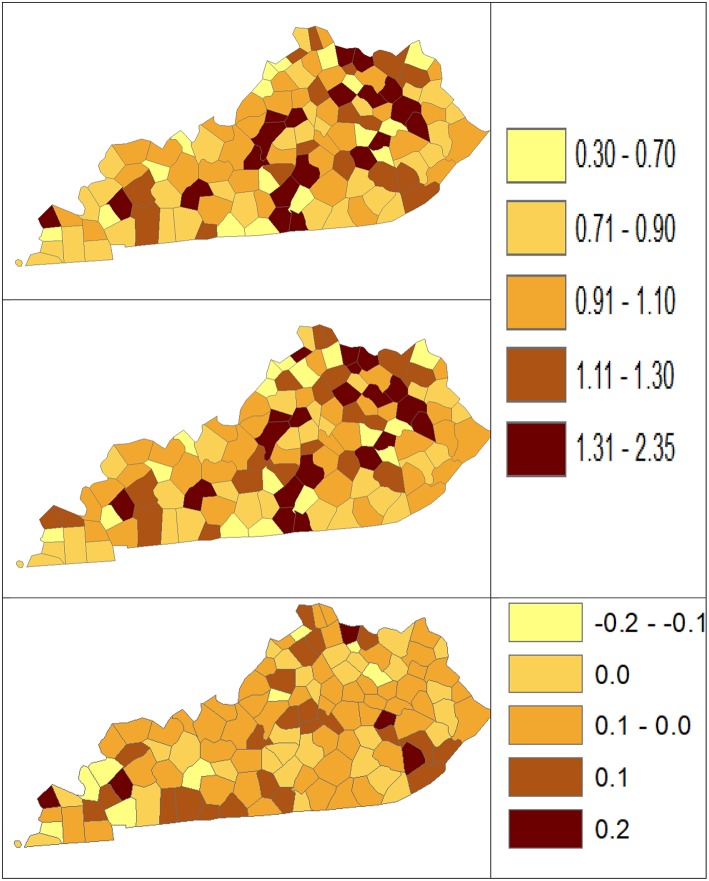
Rate Ratio estimated from statistical methods (Direct top vs. BCM middle) and their difference (bottom) using 2006–2010 oral cancer incidence dataset.

Using dataset released in the SEER^*^Stat software (16), we analyze the Kentucky male lung cancer and oral cancer (both genders) incidence data (Data has been provided in the Supplementary Material section) to obtain model-based age adjusted rate and county-to-state RR with associated credible intervals. The state of Kentucky has the highest cancer rates for both incidence and mortality. Cigarette smoking and tobacco chewing prevalence are high, especially in the southeast area of the state which is part of the Central Appalachia region (17). Tobacco use causes many types of cancer, including cancer of lung, mouth, esophagus, throat, bladder, and pancreas (18). We calculate the age-adjusted incidence rates of lung cancer (male only) and oral cancer (both genders) for the 5-year period between 2006 and 2010. These two cancer sites are selected to represent a more common and a rare cancer, respectively. The lung cancer state rate is 126.94 per 100,000 and the rates vary considerably among the 120 counties, from 57.01 to 207.21 (Figure 1, Direct), resulting in the county-to-state RR between 0.45 and 1.63 (Figure 2, Direct). There is also a spatial pattern, with higher rates in the southeast mountain area, and lower rates in the north and central areas. The oral cancer state rate is 13.12 and the county rates vary between 3.96 and 30.80 (Figure 3, Direct). The county to state RR range between 0.30 and 2.35 (Figure 4, Direct).

The population n_ij_ and number of cases D_ij_ in each region and age strata are read in the matrix of *I* (region) rows and *J* columns for each age specific count, with each region associated with a unique index (FIPS code). For each region and age stratum, the expected number of cases *e*_*ij*_ is computed using reference rates for the disease incidence: $e_{ij}=n_{ij}\frac{\overset{I}{\underset{i=1}{\sum }}D_{ij}}{\overset{I}{\underset{i=1}{\sum }}n_{ij}}$. To fit the joint model to obtain posterior mean and distribution of the relative risk λ_*ij*_, observed count and expected count with adjacency matrix are entered into the MCMC algorithm. The adjacencies for the CAR prior distribution are computed from the county or state maps using R functions in library *maps, maptools*, and library *spdep*. The program used for conditional autoregression model analysis is self-written MCMC algorithm function based on BCM using the R language (19).

## Results

Table 4 shows the point and interval estimates of AARs and RR using all four methods, for the top, middle, and lowest 5 RR of lung cancer incidence data in Kentucky counties. The interval estimates showed shrinkage from the Direct to the Overlap and to the Spatial method. By contrast, the BCM method yields different point estimates but a wider interval length as BCM incorporates the uncertainty from spatial random effect, so the interval estimate for BCM is wider. Overall, BCM provides a smoothing effect by considering the neighborhood information for the point estimates of relative risk estimates, especially for the regions with small counts. The point estimates of the AAR from BCM model algorithm are close to those from the Direct estimate (Table 4): the difference in overall rate is 0.02 and mean difference in the AAR of all regions is 1.21. The mean rate ratio difference between two methods is also minimal (0.01). However, the interval length of BCM algorithm is greater than that is calculated from the “Direct” method, for both rate and RR.

**Table 4:** Age-adjusted rate and rate ratio in top, middle, and bottom five counties based on 2006–2010 lung cancer incidence rate in Kentucky.

	**KY Counties**	**5-year population**	**Rate**								**Rate**							
			**Point estimate**				**95% Confidence interval length**				**Point estimate**				**95% Confidence interval length**			
			**Direct**	**Overlap**	**Spatial**	**BCM**	**Direct**	**Overlap**	**Spatial**	**BCM**	**Direct**	**Overlap**	**Spatial**	**BCM**	**Direct**	**Overlap**	**Spatial**	**BCM**
Top	Martin	36,297	182.625	182.625	182.625	178.942	108.940	105.321	105.321	143.209	1.439	1.439	1.439	1.411	0.860	0.827	0.795	1.131
	Menifee	16,060	191.103	191.103	191.103	198.536	141.660	133.789	133.789	189.948	1.506	1.506	1.506	1.565	1.118	1.053	1.023	1.494
	Knox	76,901	201.330	201.330	201.330	201.274	66.527	65.066	65.066	90.340	1.586	1.586	1.586	1.587	0.527	0.510	0.533	0.716
	Floyd	97,745	204.490	204.490	204.490	215.446	58.036	56.888	56.888	82.871	1.611	1.611	1.611	1.699	0.461	0.443	0.480	0.660
	Perry	70,952	207.208	207.208	207.208	215.165	69.607	68.023	68.023	96.439	1.632	1.632	1.632	1.697	0.552	0.532	0.560	0.769
Medium	Clinton	25,266	133.002	133.002	133.002	135.443	93.542	88.857	88.857	121.322	1.048	1.048	1.048	1.068	0.738	0.698	0.618	0.960
	Trigg	34,715	133.064	133.064	133.064	132.178	71.151	67.673	67.673	93.474	1.048	1.048	1.048	1.042	0.562	0.531	0.453	0.742
	Laurel	142,606	133.429	133.429	133.429	133.534	41.568	40.770	40.770	55.472	1.051	1.051	1.051	1.053	0.330	0.320	0.259	0.438
	Rockcastle	41,997	133.874	133.874	133.874	132.298	72.594	69.784	69.784	92.599	1.055	1.055	1.055	1.044	0.573	0.549	0.465	0.732
	Grayson	64,483	133.918	133.918	133.918	134.879	57.631	55.879	55.879	79.002	1.055	1.055	1.055	1.063	0.456	0.438	0.362	0.625
Low	Robertson	5,693	57.010	57.010	57.010	64.576	132.487	101.856	101.856	162.14	0.449	0.449	0.449	0.509	1.044	0.802	0.798	1.276
	Shelby	98,522	83.601	83.601	83.601	84.731	39.350	38.175	38.175	55.414	0.659	0.659	0.659	0.668	0.311	0.300	0.291	0.436
	Allen	48,168	95.687	95.687	95.687	98.046	56.487	54.081	54.081	79.390	0.754	0.754	0.754	0.773	0.446	0.425	0.395	0.629
	Boone	283,630	95.868	95.868	95.868	90.411	28.948	28.513	28.513	36.985	0.755	0.755	0.755	0.720	0.230	0.224	0.213	0.294
	Fayette	707,535	96.838	96.838	96.838	95.238	17.192	17.028	17.028	23.212	0.763	0.763	0.763	0.751	0.138	0.131	0.145	0.188

The Bayesian disease-mapping analyses for lung cancer data are presented in Figures 1, 2, which displayed the point estimate of the age-adjusted mortality rate, the rate ratio, and the difference between the direct and BCM methods (BCM minus direct) for each area based on 2006–2010 lung cancer incidence dataset. BCM provides a “smoothing” effect for the point estimates. Specifically, in eastern Kentucky, estimated region-specific AARs are higher (shaded in a darker color) based on both the direct and BCM methods. In the difference maps, the eastern Kentucky is shown in a lighter shade, meaning BCM produces lower estimates toward an overall smoothing average. By contrast, there are more areas with lower rate estimates (shaded in lighter color) in the western regions after controlling for spatial correlations. However, overall, BCM maintains “appearance” of the direct estimation while producing “smoothing” effect for the regions with extreme (high or low) rates by considering the neighborhood information.

Table 5 shows the point and interval estimates of AARs and RR using all four methods, for the top, middle, and lowest 5 RR of oral cancer incidence data in Kentucky counties. Similar to the lung cancer example, the oral cancer overall rate and region-specific AAR (and the rate ratios) yielded from BCM model are similar to the direct estimates. Fewer extreme rates are shown in the map (Figures 3, 4) produced from BCM model as compared to the map from direct estimates. The difference between the BCM and the direct estimates (bottom maps in both Figures 3, 4) shows an opposite distribution pattern, indicating the smoothing effect of the BCM method. In terms of variation, BCM method provides a wider interval than the Direct estimates for both rate and rate ratio estimates (Table 5).

**Table 5:** Age-adjusted rate and rate ratio in top, middle, and bottom five counties based on 2006–2010 oral cancer incidence rate in Kentucky.

	**KY Counties**	**5-year population**	**Rate**								**Rate ratio**							
			**Point estimate**				**Interval length**				**Point estimate**				**Interval length**			
			**Direct**	**Overlap**	**Spatial**	**BCM**	**Direct**	**Overlap**	**Spatial**	**BCM**	**Direct**	**Overlap**	**Spatial**	**BCM**	**Direct**	**Overlap**	**Spatial**	**BCM**
Top	Caldwell	64,667	21.911	21.911	21.911	20.307	22.892	20.994	20.994	26.400	1.670	1.670	1.670	1.548	1.750	1.595	1.595	2.021
	Cumberland	34,655	22.116	22.116	22.116	21.002	31.740	27.884	27.884	36.400	1.685	1.685	1.685	1.601	2.424	2.122	2.122	2.784
	Clinton	50,824	22.532	22.532	22.532	23.081	25.728	23.219	23.219	33.200	1.717	1.717	1.717	1.761	1.966	1.766	1.766	2.538
	Magoffin	66,458	22.640	22.640	22.640	22.877	23.649	21.778	21.778	30.900	1.725	1.725	1.725	1.744	1.808	1.655	1.655	2.352
	Bracken	42,385	30.797	30.797	30.797	28.983	35.614	32.760	32.760	42.100	2.347	2.347	2.347	2.209	2.722	2.491	2.491	3.216
Middle	Garrard	84,212	12.615	12.615	12.615	11.934	16.087	14.602	14.602	18.500	0.961	0.961	0.961	0.910	1.229	1.111	1.111	1.438
	Letcher	122,330	12.619	12.619	12.619	12.081	12.968	11.964	11.964	15.800	0.962	0.962	0.962	0.921	0.992	0.909	0.909	1.216
	Menifee	32,140	12.740	12.740	12.740	12.510	27.353	22.691	22.691	31.413	0.971	0.971	0.971	0.954	2.087	1.728	1.728	2.356
	Johnson	116,617	12.924	12.924	12.924	13.661	12.976	11.905	11.905	17.300	0.985	0.985	0.985	1.041	0.992	0.905	0.905	1.324
	Jefferson	3,647,412	12.930	12.930	12.930	12.733	2.270	2.243	2.243	3.0100	0.985	0.985	0.985	0.971	0.187	0.156	0.156	0.248
Bottom	Wolfe	36,562	3.961	3.961	3.961	4.240	16.096	11.126	11.126	15.500	0.302	0.302	0.302	0.324	1.228	0.848	0.848	1.170
	Hancock	42,757	4.248	4.248	4.248	3.910	15.794	12.102	12.102	14.100	0.324	0.324	0.324	0.298	1.205	0.922	0.922	1.091
	Trimble	44,351	5.384	5.384	5.384	6.020	15.950	12.256	12.256	18.400	0.410	0.410	0.410	0.459	1.217	0.934	0.934	1.407
	Carlisle	25,629	5.833	5.833	5.833	5.760	23.207	16.276	16.276	20.700	0.445	0.445	0.445	0.439	1.770	1.240	1.240	1.582
	Allen	98,485	5.988	5.988	5.988	5.200	10.912	9.632	9.632	11.500	0.456	0.456	0.456	0.397	0.833	0.733	0.733	0.875

**Figure 5:**
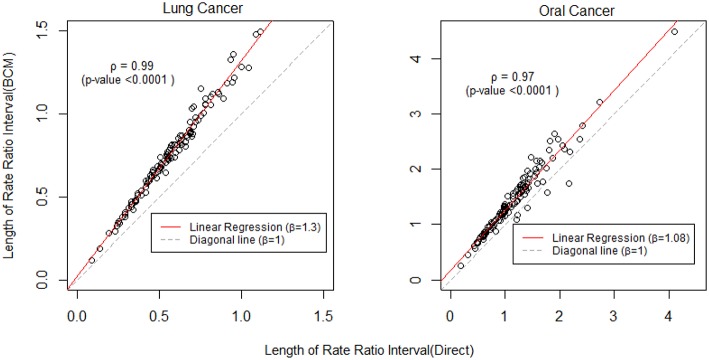
The comparison of rate ratio interval length between Direct method and BCM model (Before standardization). β is the slope for regression and ρ is the bivariate correlation.

**Figure 6:**
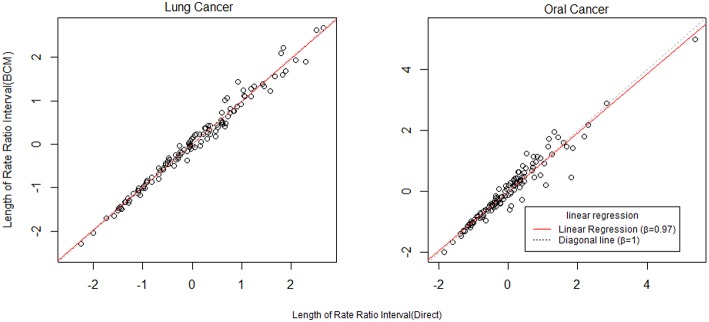
The comparison of rate ratio interval length between Direct method and BCM model (After standardization). β is the slope for regression.

To further compare the direct method and BCM model, we plot the interval length of RR between direct method and BCM model for both lung and oral cancer datasets (Figure 5). In both datasets, there is a remarkably high correlation between direct and BCM estimation. For lung cancer dataset with larger number of events, there is 30% difference in the length of RR intervals between Direct and BCM model, and on average BCM yields wider intervals as compared to the direct estimation. For oral cancer dataset, the difference in length of RR interval is < 10% between direct and BCM estimation. To make a valid comparison between the Direct and the BCM estimates on the common scale, we first standardized the interval length for RR from each method into z-scores by subtracting the mean and dividing by the standard deviation. Secondly, we applied the regression technique based on iterated re-weighted least squares (IRLS) with reweighted observations according to their absolute residuals. After standardization, there is almost a perfect correlation (99%) between two methods, the distributions of the interval length of the rate ratio are very close for both datasets (Figure 6). In other words, the county with the widest CI from the Direct method also has the widest CI from BCM estimation.

**Figure 7:**
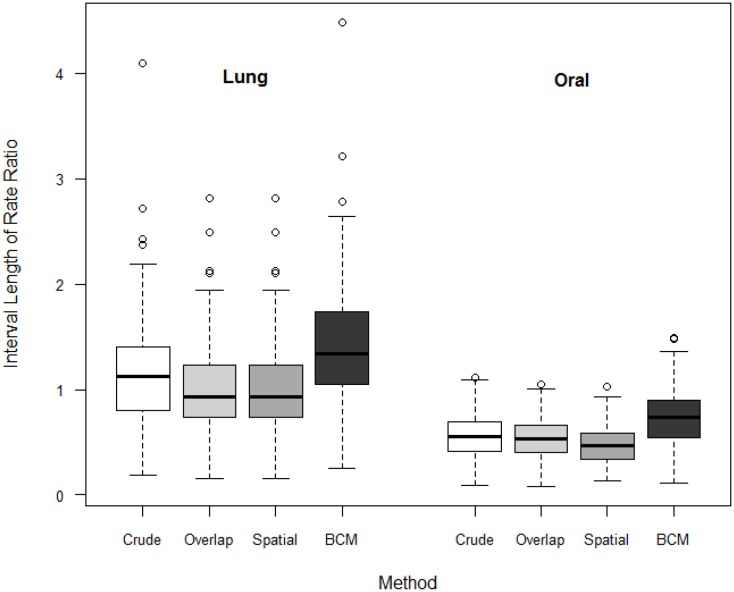
Boxplot of lengths of 95% CI for Rate Ratio by cancer site (oral or lung cancer) and method.

We compare the interval length estimates across four different methods (Figure 7). The interval length for the rate ratio shrinks from the Direct method, to the Overlap method and to Spatial method in both oral and lung cancer analyses. BCM method produces the widest CI for rate ratio in both oral and lung cancer datasets. The median rate ratio tends to be greater with BCM model as compared to the other methods.

## Discussion

In this study, the BCM method is implemented to analyze the Kentucky male lung cancer and oral cancer (both genders) incidence data acquired from the NCI SEER program, with the goal of obtaining the model-based age adjusted rate and county-to-state RR with associated credible intervals by properly taking into consideration the spatial correlation patterns. BCM allows for incorporating both uncorrelated and correlated spatial heterogeneity according to an existing neighborhood structure. Among the different methods, the Overlap and Spatial methods yield the same point estimates of rate or RR, only the interval estimates show shrinkage from the Direct method, to the Overlap and the to the Spatial method. By comparison, the proposed BCM method produces the similar point estimates for rate and RR, but since this method incorporates the uncertainty from the spatial random effect, this leads to a much wider 95% CI for the RR.

**Figure A1:**
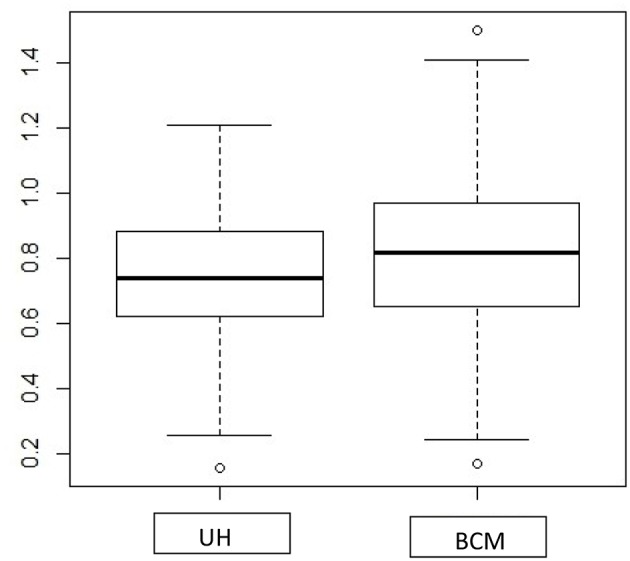
Boxplot of rate ratio CI width for comparing UH and BCM model using oral cancer incidence dataset.

Both the Spatial method and BCM consider the spatial correlation for estimating a CI for RR. For both datasets, BCM produces a much wider 95% CI for the RR, which is predominantly larger for lung cancer (higher count of events) as compared to oral cancer (small count of events) data, while the Spatial method has a shrinkage effect for the interval estimates in lung cancer data but not for oral cancer data. Unfortunately, there is little literature on the difference in interval width for comparing BCM methods to non-spatial methods. The exception is Best et al. (20) where they cite CV (coefficient of variation) for different methods but do not assess the addition of a spatial effect. One obvious reason is that two approaches have different assumptions about the form of spatial structures—the spatial method hypothesizes a parametric exponential distribution for spatial correlation structure, and the convolution model estimates spatial correlation by assuming a conditional autoregressive (CAR) prior distribution. In addition, the 95% credible interval estimated from BCM is formed by Monte Carlo draws from the posterior distribution, which tends to include a random noise component in the interval estimation, and could potentially lead to a greater CI as compared to the numerical approximation as is defined in the spatial methods. As a side interest, we also compared the length of 95% CI in RR produced from UH model and BCM model. The boxplot (Figure A1) shows the UH and BCM model widths of 95% CIs for the relative risk for Kentucky counties: the overall width is greater for BCM model.

The main advantage of using BCM is to allow flexible modeling of spatial correlation in a natural way by including uncorrelated (UH) and correlated (CH) spatial heterogeneity. Secondly, the posterior sampling based on Monte Carlo simulation tends to be more accurate than the numerical approximation, and the distributions of all the parameter estimates and model information can be obtained. Further, simulation studies have shown that BCM outperforms other methods (non-parametric smoothing methods, marginal mixture models, and full Bayes models etc.) in the analysis of small area disease incidence data with respect to overall recovery of true risk (6).

As a limitation, the data example used in this study is specifically on cancer surveillance. A larger sample could be used to illustrate the application of the methodology in a broader area in public health. More practical applications will be needed to further evaluate the performance of Bayesian convolution model and demonstrate its effectiveness. Alternatively, approximate Bayesian Inference for Latent Gaussian Models can be also obtained using Integrated Nested Laplace Approximations (INLA) (6) which can yield improved computational efficiency.

Bayesian approaches to statistical problems have gained popularity in various fields, such as epidemiology, medical and public health. Most government sources hold publicly accessible aggregated health data due to confidentiality requirements. The resulting count data, usually available at county or postal/census region level, can yield important insights into the general spatial variation of disease in terms of incidence or prevalence. However, the novel application of spatial methodology is less well-recognized in this area and it is expected to improve the efficiency of analysis of clustering effect. Bayesian convolution model (BCM) is the fundamental strategy that can incorporate the uncorrelated and correlated spatial heterogeneity effect, the extension of this model can further take into account the unobserved confounding variables that have a spatial expression over the course of the study or conduct the longitudinal type analysis. A potential future direction in this research is to check the predictive validity through simulation study or bootstrap cross-validation, so that the method can be promoted for broader planning applications. Another future research focus of this work include the inclusion of clinical and registry-level analysis, as well as population level analyses resulting from cancer registry data, the data on health service utilization, and clinical trials.

## Author Contributions

YJ developed the R program, provided analysis on the data example and results, and drafted the manuscript. AL advised the design and development of the method. LZ and EF provided guidance in developing the new method and summarized the existing methods. LZ also developed the simulation study. All authors reviewed and revised the manuscript and approved the final version.

### Conflict of Interest Statement

The authors declare that the research was conducted in the absence of any commercial or financial relationships that could be construed as a potential conflict of interest.
